# Effects of Levosimendan in Patients with Severe Mitral Insufficiency and Left Ventricular Dysfunction Undergoing Transcatheter Edge-to-Edge Repair: A Systematic Review and Meta-Analysis

**DOI:** 10.3390/jcdd13010040

**Published:** 2026-01-09

**Authors:** Stephanie Gladys Kühne, Andrea Patrignani, Simon Wölbert, Eva Harmel, Damyan Penev, Sebastien Elvinger, Mauro Chiarito, Philip W. J. Raake, Dario Bongiovanni

**Affiliations:** 1 Department of Internal Medicine I, Cardiology, University Hospital Augsburg, University of Augsburg, 86156 Augsburg, Germany; 2 Department of Cardiovascular Medicine, Humanitas Clinical and Research Center IRCCS and Humanitas University, 20089 Rozzano, Milan, Italy

**Keywords:** Levosimendan, heart failure, TEER, transcatheter edge-to-edge repair, systematic review, meta-analysis

## Abstract

Severe mitral regurgitation (MR) is one of the most common valvular heart diseases and is frequently associated with advanced left ventricular (LV) systolic dysfunction. Transcatheter edge-to-edge repair (TEER) offers effective symptom relief but may induce abrupt hemodynamic changes leading to afterload mismatch and acute LV failure. Levosimendan may help mitigate this complication by improving contractility, yet evidence supporting its use in this setting is scarce. Therefore, the aim of this study was to systematically evaluate the evidence on the effects of Levosimendan in patients with severe MR and LV dysfunction undergoing TEER. We performed a comprehensive search of PubMed, Embase, Scopus, and Google Scholar. Primary outcomes were postprocedural LV ejection fraction (LVEF) and systolic pulmonary artery pressure (sPAP). Secondary outcomes included procedural success, procedure duration, and in-hospital complications. Five studies comprising 315 patients (n = 141 Levosimendan, n = 174 controls) met the inclusion criteria. Pooled analysis showed no significant difference in postprocedural LVEF between Levosimendan-treated patients and controls (mean difference 0.45%, 95% CI [−1.46–2.35] *p* = 0.65) and no significant change from baseline. Similarly, postprocedural sPAP did not differ significantly. Procedural success was higher with Levosimendan, and procedure duration was shorter. These hypothesis-generating findings highlight the need for larger, prospective randomized trials to clarify the role of Levosimendan in this setting.

## 1. Introduction

Severe mitral regurgitation (MR) represents one of the most common valvular heart diseases in industrialized countries and is frequently associated with progressive left ventricular (LV) dilatation, systolic dysfunction, and heart failure (HF) symptoms despite guideline-directed medical therapy [1]. In patients with advanced LV dysfunction and high surgical risk, transcatheter edge-to-edge repair (TEER) has emerged as an established and effective treatment option, offering symptomatic relief and reverse cardiac remodeling in both primary and functional MR [1,2,3,4,5].

However, in this frail population, TEER may be accompanied by abrupt hemodynamic changes due to the sudden elimination of the low-impedance regurgitant pathway into the left atrium. The consequent increase in LV afterload can transiently impair systolic function. This phenomenon, referred to as afterload mismatch [6,7,8], can induce acute LV failure with subsequent pulmonary congestion and frequently requires initiation of inotropic or mechanical circulatory support. While often reversible, afterload mismatch remains a clinically relevant determinant of early outcomes after TEER and may complicate periprocedural management, particularly in patients with severely reduced LV ejection fraction (LVEF) at baseline.

Among pharmacologic strategies to prevent or mitigate this hemodynamic deterioration, Levosimendan has gained attention as a potential adjunctive therapy [9]. Levosimendan acts as a calcium sensitizer and ATP-sensitive potassium channel opener, exerting combined inotropic and vasodilatory effects without increasing myocardial oxygen consumption [10]. These properties suggest that it may be well suited for periprocedural support of patients undergoing TEER, particularly in those with advanced HF and reduced contractile reserve.

Despite its increasing use in clinical practice [11], the evidence supporting Levosimendan in this setting remains limited, heterogeneous, and so far no evidence-based synthesis of the literature is currently available. Therefore, we aim to comprehensively review the totality of the existing literature of Levosimendan in patients with severe mitral insufficiency and systolic left ventricular dysfunction undergoing transcatheter edge-to-edge repair, providing the first evidence-based synthesis on this issue.

## 2. Materials and Methods

### 2.1. Comprehensive Literature Search

This systematic review and meta-analysis was designed and conducted in accordance with the Preferred Reporting Items for Systematic Reviews and Meta-Analyses (PRISMA) guidelines [12] (Supplemental Table S1). The study protocol was registered in the International Prospective Register of Systematic Reviews (PROSPERO-CRD420251229432).

### 2.2. Search Strategy

A comprehensive literature search was carried out across PubMed, Embase, Scopus, and Google Scholar databases from inception to 15 September 2025 to identify all relevant studies. Search terms included combinations of Medical Subject Headings (MeSH) and free-text keywords such as “Levosimendan” AND (“mitral regurgitation” OR “mitral insufficiency”) AND (“transcatheter edge-to-edge repair” OR “MitraClip” OR “percutaneous mitral repair”) AND (“left ventricular dysfunction” OR “heart failure”).

No language or publication date restrictions were applied. In addition, manual backward snowballing was performed by screening reference lists of all retrieved articles and relevant reviews to identify additional eligible studies. Conference abstracts were included when sufficient information could be obtained.

### 2.3. Study Selection and Eligibility Criteria

All titles and abstracts identified through the search were independently screened by two reviewers (S.K. and D.B.). Full-text evaluation was subsequently performed for potentially eligible studies according to predefined inclusion and exclusion criteria. We included randomized controlled trials, observational cohort studies, and case reports evaluating the periprocedural administration of Levosimendan in adult patients with severe MR and left ventricular dysfunction undergoing transcatheter edge-to-edge repair (TEER).

Studies were required to report at least one of the following outcomes: in-hospital mortality, afterload mismatch occurrence, left ventricular ejection fraction (LVEF) changes, systolic pulmonary artery pressure (sPAP), and major adverse cardiac events (MACE). Literature reviews and animal studies were excluded from quantitative synthesis.

Disagreements between reviewers were solved by consensus or consultation with a third investigator (S.W.). Data were extracted using a standardized form that included study design, population characteristics, pre- and postprocedural echocardiographic parameters, and clinical outcomes.

### 2.4. Risk of Bias Assessment

Risk of bias assessment was performed according to Cochrane recommendations. Risk of bias in non-randomized studies was evaluated with ROBINS-I tool [13] (Supplemental Table S2). The case report was critically appraised using the JBI Checklist for Case Reports [14] (Supplemental Table S3). All key reporting domains were fulfilled.

### 2.5. Statistics

Aggregate statistics were calculated using weighted means and pooled standard deviations based on study sample sizes. Continuous variables are presented as mean ± standard deviation, while categorical variables are expressed as counts and percentages. For data reported as median with interquartile range, means and standard deviations were estimated using the methods described by Luo et al. for means [15] and Wan et al. for standard deviations [16]. Between-group comparisons were performed using independent samples t-tests for continuous variables and Fisher’s exact tests for categorical variables.

Random-effects meta-analysis was conducted using the inverse variance method and the restricted maximum-likelihood estimator to pool mean differences for each endpoint. Heterogeneity was assessed using the I^2^ statistic and the Cochran’s Q test.

All tests were two-tailed with statistical significance set at *p* < 0.05. All statistical analyses were conducted using R statistical software (version 4.3.0).

## 3. Results

### 3.1. Literature Search Results

A total of 433 records were identified through database searches and manual reference screening (PubMed, Embase, Scopus, and Google Scholar). After removing 178 duplicates, 255 records were screened by title and abstract. Of these, 27 full-text articles were assessed for eligibility. Ultimately, five studies [17,18,19,20,21] met the inclusion criteria and were incorporated into the qualitative and quantitative synthesis.

**Figure 1:**
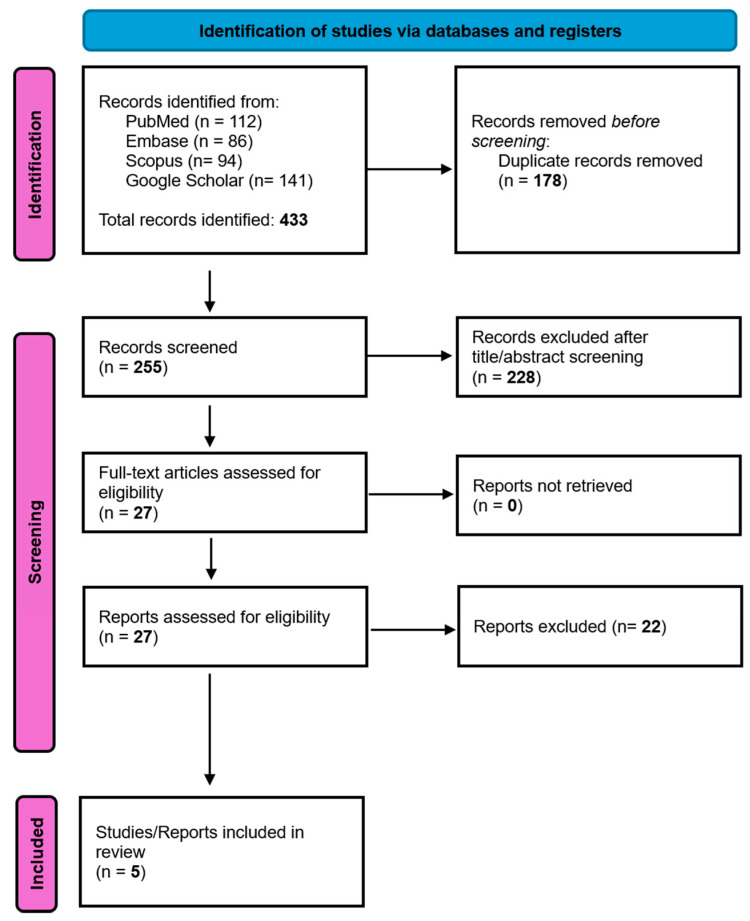
Search strategy using the PRISMA flow chart [12].

The study selection process according to PRISMA is summarized in Figure 1.

### 3.2. Study Characteristics

A total of five studies [17,18,19,20,21] published between 2016 and 2025 were included. The final data set was derived from three single- or multicenter registries, one prospective observational study, and one case report conducted across Italy, Spain, and France (Table A1). Overall, the pooled sample included 315 patients, of whom 141 received Levosimendan (L-group) and 174 served as controls (non-L group).

The included studies consistently represented high-risk populations with severe mitral regurgitation and marked left ventricular systolic dysfunction (preprocedural mean LVEF L-group 27.6 ± 6.5; non-L group 28.8 ± 6.1, *p* = 0.107) undergoing TEER. Levosimendan dosing protocols were relatively homogeneous across studies, typically involving continuous intravenous infusion (rate: 0.01 μg/kg/min) without a loading dose.

A detailed summary of the included studies covering design, sample size, key findings, and database source is presented in Table A1.

### 3.3. Population and Baseline Echocardiographic Characteristics

The mean age was comparable between groups (L-group 67.9 ± 9.8 vs. non-L group 68.6 ± 10.3 years; *p* = 0.616), and the proportion of male patients did not differ significantly (80.5% vs. 64.1%; *p* = 0.134). Preprocedurally, patients in the Levosimendan group presented with significantly more advanced heart failure symptoms, as indicated by a higher proportion of New York Heart Association (NYHA) functional classes II–IV (*p* < 0.001). Baseline renal function was similar between groups (GFR L-group 50.9 ± 22.7 vs. non-L group 52.7 ± 24.8 mL/min; *p* = 0.609, Table 1).

Preprocedural echocardiographic parameters revealed severe LV systolic dysfunction in both cohorts, with mean LVEF 27.6 ± 6.5% vs. 28.8 ± 6.1% (*p* = 0.107). LV volumes were markedly increased, and pulmonary pressures were slightly higher in the L-group (sPAP 49.7 ± 12.1 vs. 46.8 ± 14.1 mmHg; *p* = 0.049). The effective regurgitant orifice area (EROA) was significantly larger among Levosimendan-treated patients (0.49 ± 0.17 cm^2^ vs. 0.36 ± 0.19 cm^2^; *p* < 0.001); however, these data were available for only 41 and 95 patients in the L-group and non-L group, respectively. Right ventricular function was comparably reduced in both groups (TAPSE 16.8 ± 4.1 vs. 17.1 ± 4.1 mm; *p* = 0.676, Table 2).

### 3.4. Quantitative Synthesis

#### 3.4.1. Left Ventricular Ejection Fraction (LVEF)

**Figure 2:**
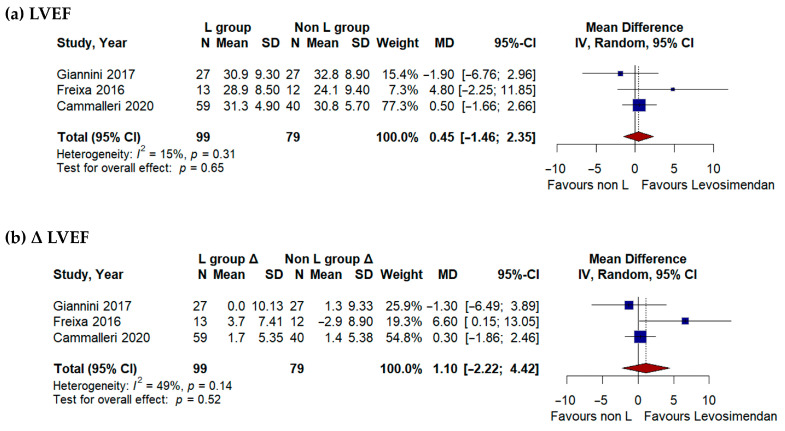
(**a**) Pooled analysis of postprocedural left ventricular ejection fraction (LVEF) comparing L-group versus non-L group. (**b**) Mean change (Δ) in LVEF from baseline to postprocedure in the Levosimendan group versus control group [17,18,21].

Three studies [17,18,21] including 178 patients reported data on postprocedural LVEF (Figure 2). The pooled analysis showed no significant difference in LVEF between patients treated with Levosimendan and controls (mean difference 0.45%, 95% CI −1.46 to 2.35; *p* = 0.65; I^2^ = 15%). Similarly, the pooled change in LVEF from baseline to postprocedure did not differ significantly between groups (mean difference 1.10%, 95% CI −2.22 to 4.42; *p* = 0.52; I^2^ = 49%).

#### 3.4.2. Systolic Pulmonary Artery Pressure (sPAP)

**Figure 3:**
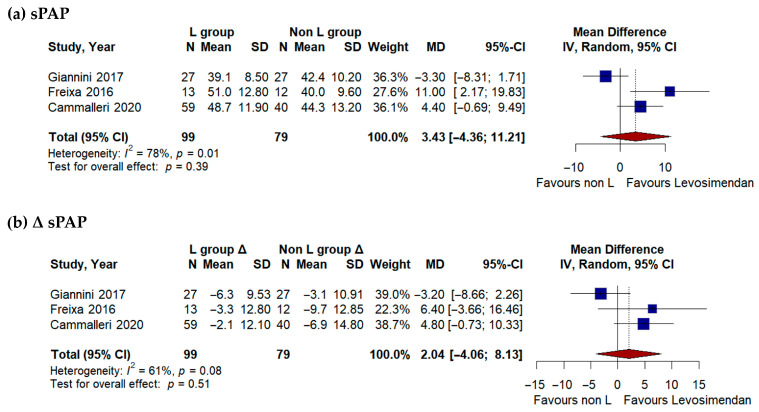
(**a**) A. Pooled postprocedural systolic pulmonary artery pressure (sPAP) comparing Levosimendan-treated and control patients. (**b**) Change (Δ) in systolic pulmonary artery pressure (sPAP) before and after TEER in Levosimendan versus control groups [17,18,21].

Three studies [17,18,21] provided data on pulmonary pressures before and after TEER (Figure 3). Pooled analysis of postprocedural sPAP values showed no statistically significant difference between the Levosimendan and control groups (mean difference 3.43 mmHg, 95% CI −4.36 to 11.21; *p* = 0.39; I^2^ = 78%). Likewise, the change in sPAP from baseline to follow-up did not reach statistical significance (mean difference 2.04 mmHg, 95% CI −4.06 to 8.13; *p* = 0.51; I^2^ = 61%).

### 3.5. Procedural and In-Hospital Outcomes

Data on procedural success were available for 154 patients, which was achieved in 100% of patients receiving Levosimendan compared with 91.0% in the control group (*p* = 0.006). The procedure duration was significantly shorter in the L-group (137.9 ± 48.2 vs. 181.6 ± 63.7 min; *p* < 0.001). Overall, in-hospital mortality was low and did not differ between groups (1.0% vs. 0.0%; *p* = 1.000).

The incidence of periprocedural complications such as pericardial tamponade, severe bleeding, acute renal failure, or sepsis was rare and comparable between groups (Table 3). New-onset atrial fibrillation occurred in 2.0% of patients in the Levosimendan group and 1.3% in controls (*p* = 1.000). Postprocedural brain natriuretic peptide (BNP) levels, available only in a limited group of patients [17,18,19,21], were numerically higher in the Levosimendan cohort but without reaching statistical significance (694.5 ± 412.3 vs. 565.5 ± 393.2 ng/mL; *p* = 0.245). The mean length of hospital stay was similar between groups (4.5 ± 3.0 vs. 4.5 ± 1.5 days; *p* = 0.961) (Table 3).

## 4. Discussion

This systematic review and meta-analysis provides the first comprehensive synthesis of the totality of the existing literature on Levosimendan in TEER patients with advanced left ventricular dysfunction. The analysis showed no significant pooled effect of Levosimendan on early LVEF or sPAP.

In cardiac surgery, preoperative administration of Levosimendan in patients with severe left ventricular dysfunction (LVEF < 35%) has shown a reduction in all-cause mortality in patients undergoing coronary artery bypass grafting [11]. These findings have prompted interest in whether similar benefits might exist in the TEER population, where acute afterload changes pose a relevant clinical challenge. Yet, clinical evidence remains limited. So far, non-randomized studies have pointed towards improved periprocedural hemodynamics and trends towards improved short-term outcomes [17,18]. The lack of prospective randomized trials underscores the need for robust evaluation of periprocedural Levosimendan therapy in this growing patient cohort.

A mechanistic rationale for Levosimendan in TEER patients with reduced systolic function is plausible, possibly involving its effect on the left-ventricular pressure–volume relationship [20]. In systolic heart failure, left-ventricular contractile performance is reduced, and filling pressures are frequently elevated. Following TEER, the sudden reduction in mitral regurgitation increases the effective afterload, which may further compromise stroke volume and cardiac output in an already impaired left ventricle [20]. Afterload mismatch can be observed in the form of an acute reduction in LVEF [8] and/or as acute LV remodeling, and it is discussed as a possible risk factor for poorer clinical outcome [6]. Levosimendan may mitigate these effects through two possible pathways. The drug acts as a calcium sensitizer [10] providing an inotropic effect with minimal additional energetic demand and may improve systolic pressure generation under the modified loading conditions in TEER. In parallel, Levosimendan activates ATP-dependent potassium channels in vascular smooth muscle, resulting in arterial and venous vasodilatation [22,23]. The resulting reduction in systemic vascular resistance lowers afterload and facilitates left-ventricular ejection.

Moreover, the clinical heterogeneity among TEER candidates [24], including broad variability in LV size, right-ventricular involvement, or pulmonary hypertension, indicates that patient-specific hemodynamics are likely to modulate the response to Levosimendan. Emerging digital-twin technologies [25] might in the future enable a patient-specific simulation of the hemodynamic changes expected after TEER. This personalized-medicine approach may help to identify patients at highest risk for afterload mismatch and determine which patients are likely to benefit from Levosimendan.

Interestingly, the potential beneficial effects of Levosimendan have also been indicated in acute myocardial infarction patients, suggesting favorable properties under ischemic and reperfusion conditions [26]. Data in an ischemia–reperfusion injury heart model point towards an increase in coronary flow and a reduction in arrhythmias [27]. The underlying mechanisms may also play a role in the periprocedural resilience of severely impaired ventricles undergoing TEER.

The findings of this systematic review and meta-analysis should be interpreted in light of several considerations. First, the available evidence is derived from relatively small observational studies, as randomized controlled trials in this setting are currently lacking. Consequently, some degree of residual confounding or selection bias cannot be excluded, particularly because Levosimendan tends to be used in higher-risk patients in clinical practice. This possible preselection bias could mask a beneficial effect of Levosimendan, resulting in similar outcomes between groups. Second, the overall sample size was modest (315 patients), and not all studies reported all outcomes of interest, especially periprocedural complications. Furthermore, data on long-term clinical outcomes such as long-term mortality, rehospitalization for heart failure, and sustained ventricular remodeling were rarely reported and could not be meta-analyzed. Finally, the analysis relied on aggregated published data and not on individual patient-level data, which precludes more granular analyses of dose, timing and duration of Levosimendan, of center-specific practice patterns, and of potential effect modifiers. Dosing regimens differed between the studies. While for example Giannini et al. [17] administered Levosimendan 6–12 h before the intervention continuously over 24 h before the intervention (rate 0.01 μg/kg/min; no loading dose), Frexia et al. [18] and Cammalleri et al. [21] started Levosimendan 1 h before the procedure (rate 0.01 μg/kg/min; no loading dose). Overall, the present findings should be regarded as exploratory and hypothesis-generating rather than definitive.

Our analysis did not show early improvement of echocardiographic parameters but points towards a higher procedural success in Levosimendan-treated patients, although based on a limited patient number, making it a plausible adjunct in selected high-risk patients. Prospective randomized trials with long-term functional and survival endpoints are urgently needed to further investigate the use of Levosimendan in this setting.

## 5. Conclusions

This systematic review and meta-analysis provides the first systematic insight into the potential effects of periprocedural Levosimendan in patients with systolic heart failure undergoing TEER. The findings are hypothesis-generating and highlight the need for larger, prospective randomized trials to clarify the role of Levosimendan in this setting.

## Figures and Tables

**Table 1:** Baseline characteristics. NYHA—New York Heart Association Functional Class; GFR (mL/min)—Glomerular Filtration Rate * statistical significance.

Variable	L-Group	Non-L Group	*p*-Value
Age (years)	67.9 ± 9.8 (n = 82)	68.6 ± 10.3 (n = 134)	0.616
Male (sex)	33/41 (80.5%)	25/39 (64.1%)	0.134
NYHA II	2/67 (3.0%)	27/101 (26.7%)	
NYHA III	47/67 (70.1%)	61/101 (60.4%)	
NYHA IV	18/67 (26.9%)	13/101 (12.9%)	0.001< *
GFR (mL/min/1, 73 m^2^)	50.9 ± 22.7 (n = 69)	52.7 ± 24.8 (n = 122)	0.609

**Table 2:** Preprocedural echocardiographic parameters. LVEF—left ventricular ejection fraction; TAPSE (mm)—tricuspid annular plane systolic excursion; sPAP (mmHg)—systolic pulmonary artery pressure; LVEDV (mL)—left ventricular end-diastolic volume; EROA (cm^2^)—effective regurgitant orifice area; LVEDD (mm)—left ventricular end-diastolic diameter; LVESD (mm)—left ventricular end-systolic diameter; * statistical significance.

Variable	L-Group	Non-L Group	*p*-Value
LVEF (%)	27.6 ± 6.5 (n = 141)	28.8 ± 6.1 (n = 174)	0.107
TAPSE (mm)	16.8 ± 4.1 (n = 69)	17.1 ± 4.1 (n = 122)	0.676
sPAP (mmHg)	49.7 ± 12.1 (n = 141)	46.8 ± 14.1 (n = 174)	0.049
LVEDV (mL)	255.6 ± 88.0 (n = 42)	229.0 ± 67.0 (n = 95)	0.086
EROA (cm^2^)	49.0 ± 17.0 (n = 41)	36.0 ± 19.0 (n = 95)	0.001< *
LVEDD (mm)	69.0 ± 6.6 (n = 13)	69.4 ± 7.5 (n = 12)	0.889
LVESD (mm)	57.2 ± 9.4 (n = 13)	55.9 ± 8.0 (n = 12)	0.712

**Table 3:** Comparison of procedural and in-hospital outcomes between L-group and non-L group undergoing TEER.

Variable	L-Group	Non-L Group	*p*-Value
Procedural success	87/87 (100.0%)	61/67 (91.0%)	0.006
In-hospital mortality	1/100 (1.0%)	0/79 (0.0%)	1.000
Procedural time (min)	137.9 ± 48.2 (n = 40)	181.6 ± 63.7 (n = 39)	0.001<
Pericardial tamponade	1/28 (3.6%)	0/27 (0.0%)	1.000
Severe bleeding	1/28 (3.6%)	2/27 (7.4%)	0.611
New onset atrial fibrillation	2/100 (2.0%)	1/79 (1.3%)	1.000
Sepsis	0/28 (0.0%)	2/27 (7.4%)	0.236
Acute renal failure	0/28 (0.0%)	1/27 (3.7%)	0.491
BNP (ng/mL)	694.5 ± 412.3 (n = 27)	565.5 ± 393.2 (n = 27)	0.245
Length of hospital stay (days)	4.5 ± 3.0 (n = 40)	4.5 ± 1.5 (n = 39)	0.961

## Data Availability

Primary data are available upon reasonable request.

## Supplementary Materials

The following supporting information can be downloaded at: https://www.mdpi.com/article/10.3390/jcdd13010040/s1, Table S1: PRISMA (preferred Reporting Items for Systematic Review and Meta-Analysis; Table S2: Risk of bias analysis according to ROBINS-I; Table S3: Risk of bias assessment for case reports using JBI Critical Appraisal Checklist for Case Reports.

## Abbreviations

The following abbreviations are used in this manuscript:

EROA	Effective Regurgitant Orifice Area
GFR	Glomerular Filtration Rate
HF	Heart Failure
LV	Left Ventricular
LVEDD	Left Ventricular End-Diastolic Diameter
LVEDV	Left Ventricular End-Diastolic Volume
LVEF	Left Ventricular Ejection Fraction
LVESD	Left Ventricular End-Systolic Diameter
MACE	Major Adverse Cardiac Event
MR	Mitral Regurgitation
NYHA	New York Heart Association Functional Class
PRISMA	Preferred Reporting Items for Systematic Reviews and Meta-Analyses
sPAP	Systolic Pulmonary Artery Pressure
TAPSE	Tricuspid Annular Plane Systolic Excursion
TEER	Transcatheter Edge-to-Edge Repair

## Appendix A

**Table A1:** Synthesis of the totality of the current existing literature on the issue.

Author	Title	Year	Country	DOI	Study Design	L-Group (n)	Non-L Group (n)	Key Message	Database
Giannini [17]	Effects of Levosimendan in patients with severe functional mitral regurgitation undergoing MitraClip implantation	2017	Italy	10.2459/JCM.0000000000000537	Prospective registry	27	27	Levosimendan as an adjunctive therapy to MitraClip implantation offers further therapeutic advantages in patients with advanced heart failure by improving systolic right ventricle function.	PubMed
Freixa [18]	Levosimendan as an adjunctive therapy to MitraClip implantation in patients with severe mitral regurgitation and left ventricular dysfunction	2016	Spain	doi.org/10.1016/j.ijcard.2015.09.056	Observational study without randomization	13	12	Adjunctive use of Levosimendan in patients with severe MR and LV dysfunction undergoing MitraClip repair was well tolerated and associated with a lower incidence of hemodynamic deterioration after MitraClip implantation.	PubMed
Koutsoukis [19]	MitraClip Implantation for Functional Mitral Regurgitation with Coaptation Gap Facilitated by Levosimendan Treatment	2020	France	10.1016/j.jaccas.2020.04.017	Case report	1	0	Use of Levosimendan to facilitate an MC procedure in a patient with unfavorable anatomy.	PubMed
Cammalleri [21]	Acute effects of Levosimendan on myocardial function in patients with severe mitral regurgitation and left ventricular dysfunction undergoing MitraClip repair	2020	Italy	doi.org/10.1093/ehjci/jez319.152	Single center	59	40	In MitraClip patients with severe reduction in LVEF, Levosimendan improved hemodynamic outcome, increasing myocardial contractility during and early after procedure.	Google Scholar
Angelini [20]	Afterload mismatch after transcatheter edge-to-edge repair in functional mitral regurgitation: A propensity-score matched analysis	2025	Italy	10.1111/eci.70040	Multicenter case–control study	41	95	Patients pretreated with Levosimendan were less likely to suffer from afterload mismatch.	PubMed
